# Common Mental Disorder and Associated Factors among Women Attending Antenatal Care Follow-Up in North Wollo Public Health Facilities, Amhara Region, Northeast Ethiopia: A Cross-Sectional Study

**DOI:** 10.1155/2024/8828975

**Published:** 2024-03-21

**Authors:** Amanuel Addisu, Henok Kumsa, Seteamlak Adane, Gedefaw Diress, Aragaw Tesfaye, Mulugeta Wodaje Arage, Kendie Mekuria, Solomon Moges, Getasew Mulat Bantie, Amare Alemu Melese, Lebeza Alemu Tenaw

**Affiliations:** ^1^Department of Public Health, College of Medicine and Health Science, Injibara University, Injibara, Ethiopia; ^2^Department of Midwifery, School of Midwifery, College of Health Science, Woldia University, Woldia, Ethiopia; ^3^Department of Public Health, School of Public Health, College of Health Science, Woldia University, Woldia, Ethiopia; ^4^Department of Public Health, College of Health Science, Debre Markos University, Debre Markos, Ethiopia; ^5^School of Medicine, College of Health Science, Woldia University, Woldia, Ethiopia; ^6^College of Health Science, Woldia University, Woldia, Ethiopia; ^7^Amhara National Regional State Public Health Institute, Bahir Dar, Ethiopia; ^8^Ethiopian Public Health Institute, Addis Ababa, Ethiopia

## Abstract

**Introduction:**

Common mental health disorders (CMD) during pregnancy are a public health concern because of the implications for the mother and infant's health during pregnancy and after birth. The prevalence and factors related to common mental disorders vary globally. Therefore, this study assessed the magnitude and factors associated with common mental disorder among pregnant women attending ANC follow-up in North Wollo Zone, Northeast Ethiopia.

**Methods:**

An institutional-based cross-sectional study was conducted in North Wollo, Amhara Region, Northeast Ethiopia. A multistage sampling technique was used to select 777 study participants. The common mental disorder was assessed by using SRQ-20. Data were analyzed using Statistical Package for the Social Sciences (SPSS) version 23. Logistic regression analysis was done to identify the independent variables associated with common mental disorders. Independent variables with a *p* value less than 0.05 were considered significantly associated with CMD.

**Results:**

The magnitude of CMD was 18.1% (95% CI: 15.5, 21.0). Factors significantly associated with CMD were the educational level of participants (AOR = 0.17, 95% CI: 0.06, 0.48), husband's educational status (AOR = 11.13, 95%: 4.18, 29.66), unplanned pregnancy (AOR = 2.54, 95% CI: 1.26, 5.09), self-reported complication on the current pregnancy (AOR = 0.11, 95% CI: 0.05, 0.21), self-reported complication during the previous delivery (AOR = 3.38, 95% CI: 1.39, 8.18), undernutrition (AOR = 2.19, 95%: 1.26, 3.81), high psychosocial risk (AOR = 20.55, 95% CI: 9.69, 43.59), having a legal issue (AOR = 2.06, 95%: 1.12, 3.79), and relationship problem (AOR = 7.22, 95% CI: 3.59, 14.53). *Conclusions and Recommendation*. One in five pregnant women has common mental disorder. Educational status of the participants and their spouses, unplanned pregnancy, self-reported complication during current and previous pregnancy, psychosocial risk, and legal and relationship problems were the main determinants of common mental disorders. Therefore, screening pregnant women for mental disorders and provision of necessary mental health services are recommended to minimize the adverse health outcome of CMD during pregnancy.

## 1. Introduction

Mental disorders are the major public health concerns during pregnancy, affecting the mother and child [1]. Globally, approximately 10% of pregnant women experience a mental disorder, with around 12% of women experiencing depression and 13% experiencing anxiety, and sometimes, women may experience both [2].

Common mental disorder (CMD) is a general term for mental health conditions, including depression, anxiety, and medically unexplained physical symptoms [3]. Common mental disorder is also often reported in low-middle-income countries (LMICs), from particularly women. The burden is higher in LMICs as CMD ranges from 12 to 43% during pregnancy and 19.8% after they gave birth in LMICs [4–6]. However, CMD in LMICs is an underestimated public health problem causing a significant contribution to maternal and infant morbidity and mortality [7].

The presence of CMD at the time of pregnancy could cause a decrease in seeking antenatal services, which may increase the chance of complicated delivery and poor child health outcomes such as low birth weight, preterm delivery, increased neonatal and infant mortality, and postnatal psychosis which lead to subsequent behavioral, emotional, and cognitive problems [1, 8–10]. Additionally, CMD during pregnancy has been associated with substance abuse and elevated maternal cortisol, which are predictors of adverse neonatal outcomes, impaired cognitive development, and future behavioral problems [11, 12]. Furthermore, poor maternal mental health is associated with negative social, economic, and psychological consequences for the individuals, their children, their families, and the community [13].

Factors associated with CMD among pregnant women in LMICs include being illiterate; being single, widowed, divorced, or separated; having an unsupportive partner; physical violence; verbal abuse; alcohol use; having more than three children; having HIV/AIDS; and death of a loved one [14–17]. Additionally, studies revealed that factors such as unplanned pregnancy, gestational age and bleeding, presence of other medical problems before conception, absence of support, and low socioeconomic status were associated with antenatal CMD [18–20].

Ethiopia is one of the LMICs with increased rates of mental health problems and antenatal CMD ranging from 9.2 to 35.8% [18, 21]. There is a scarcity of studies done at different health facilities in Ethiopia as the previous studies focused on assessing CMD in a single institution which may only address some women seeking antenatal services from the health center to the hospital level. The vast implications of antepartum mental health issues make a public health concern. Interventions to improve maternal mental health during pregnancy are important in preventive strategies against adverse health and developmental outcomes in newborns and children. Therefore, this study is aimed at assessing the magnitude and associated factors among pregnant women attending an ANC visit in North Wollo Zone public health facilities, Amhara Region, Northeast Ethiopia.

## 2. Methods

### 2.1. Study Area

This study was conducted in North Wollo Zone, 521 km away from Addis Ababa, the capital city of Ethiopia. It has sixty-eight health centers and six hospitals. According to the North Wollo Zone administration office 2020 report, around twenty-four percent of the population was women of childbearing age [22].

### 2.2. Study Design

An institutional-based cross-sectional study was conducted in North Wollo Zone public health facilities, Amhara Region, Northeast Ethiopia.

### 2.3. Source Population and Study Population

All pregnant women attending ANC visits in the North Wollo Zone health facilities, Amhara Region, Northeast Ethiopia, were the source population. The study population was pregnant women attending ANC visits in selected North Wollo Zone public health facilities, Amhara Region, Northeast Ethiopia.

### 2.4. Inclusion and Exclusion Criteria

All pregnant women at any gestational age who are attending ANC visits were included in the study, while those pregnant women who come for medical treatment including trauma were excluded.

### 2.5. Sample Size Determination

The sample size was determined using a single population proportion formula considering the prevalence of antenatal CMD (35.8%) [21], level of significance (95%) (*α* = 0.05), absolute precision or margin of error (5%), design effect of two, and nonresponse rate (5%); the final sample size became 777.

### 2.6. Sampling Method

A multistage sampling technique was employed to select study participants. From 68 health centers and six hospitals, 22 health centers and two hospitals were selected using a simple random sampling technique. The final sample was allocated proportionally to the selected public health institution based on the average number of ANC follow-ups. Then, systematic random sampling was employed to select pregnant women who are attending their antenatal visits in the selected public health facilities.

### 2.7. Study Variables and Measurements

#### 2.7.1. Dependent Variable

Antenatal common mental disorder was assessed using the interviewer-administered self-reporting questionnaire (SRQ-20). The self-reporting questionnaire is a 20-item questionnaire consisting of 20 yes/no questions about the experience of depressive, anxiety, and somatic symptoms in the last 30 days. The reliability and validity of SRQ-20 have been established in Ethiopia [23]. Those who responded “yes” six and above to the twenty questions were categorized as having antenatal CMD [24, 25].

#### 2.7.2. Independent Variables

- *Sociodemographic status variables* were age, residence, marital status, educational status, and occupational status
- *Obstetric history variables* were gravidity, parity, gestational age, history of previous pregnancy complications, current pregnancy complications, the total number of live births, history of stillbirth, and history of abortion
- *Medical history variables* were family history of psychiatric disorder and confirmed chronic medical illness
- *Substance use*: WHO ASSIST is an alcohol, smoking, and substance involvement screening test, and it was used to screen pregnant women for substance use. It is a brief screening questionnaire for psychoactive substances. It was developed by the World Health Organization (WHO) and an international team of substance use researchers as a simple screening tool for hazardous, harmful, and dependent use of alcohol, tobacco, and other psychoactive substances [26]
- *Maternal nutritional status*: mid-upper arm circumference (MUAC) was used to assess the nutritional status of pregnant mothers. MUAC of less than 23 cm was considered a sign of undernutrition. MUAC measurements of pregnant mothers were taken using an MUAC tape and recorded using a standardized checklist [27]
- *Psychosocial health*: psychosocial health during pregnancy was measured during recruitment using the Antenatal Risk Questionnaire (ANRQ). The Antenatal Risk Questionnaire (ANRQ) addresses key domains of psychosocial health associated with increased risk of perinatal mental health morbidity (e.g., depressive or anxiety disorder) and less optimal mother-infant attachment. The ANRQ can be self-completed or administered by the clinician and used during pregnancy or postnatal care. The ANRQ has 12 scored items relating to the following risk domains: mental health history; history of physical, sexual, or emotional abuse or neglect; level of practical support and emotional support from a partner; anxiety and perfectionism levels; and stressors/losses in the last year (e.g., bereavement and separation) [28]

### 2.8. Data Collection Method

Data were collected from systematically selected pregnant women who presented for ANC follow-up at the selected health facilities, using an interviewer-administered structured questionnaire, standardized and validated screening tools (SRQ-20, LTE-Q, WHO ASSIST, and ANRQ), and a data abstract sheet. MUAC measurements of pregnant mothers were taken using an MUAC tape and recorded using a standard checklist. In the data collection process, twenty-four data collectors and four supervisors participated.

### 2.9. Data Quality Assurance

Data were collected using standardized screening tools. The questionnaires were prepared in English and translated into the local language (Amharic). Training was given to data collectors and supervisors. The supervisors and principal investigator checked the collected data daily for completeness and consistency.

### 2.10. Data Analysis

The collected data were entered into EpiData 3.5.1 and exported to SPSS version 23.0 for analysis. Frequency and percentage were used for the descriptive statistics presentation, and a logistic regression model was used to identify the statistically significant variables. The statistically significant variables with *p* value < 0.2 at bivariable logistic regression were entered into the multivariable analysis. The multivariable logistic regression was used to identify statistically significant variables with *p* value < 0.05, and an odds ratio with a 95% confidence interval was computed to determine the significance level.

## 3. Result

### 3.1. Sociodemographic Characteristics of the Study Participants

The study was conducted on 777 pregnant women in selected public health institutions, with a 99.8% response rate. The mean (±SD) age of women was 28.9 ± 5.7 years. Three-fourths (585 (75.3%)) of the pregnant women were between 20 and 34 years old (with the age range of 20 to 34 years), and 613 (78.9%) were married. Above one-fifth (22.1%) of the pregnant women had a college and above education (Table 1).

### 3.2. Obstetric Characteristics of the Study Participants

More than half of the pregnant women (417 (53.7%)) were in the third trimester of pregnancy, and nearly one-third (251 (32.3%)) of the pregnant women had MUAC measurements below the normal value. Fifty-seven (11.1%) and 18 (3.5%) pregnant women had a history of abortion and stillbirth, respectively. Moreover, one hundred twenty-eight (16.5%) pregnant women had pregnancy complications. The obstetric characteristics of the study participants are presented in Table 2.

### 3.3. Psychosocial Risk of the Study Participants

Eighty-four (10.8%) pregnant women had high psychosocial risk during pregnancy, and 76 (9.8%) pregnant women had a history of being worried or miserable for more than two weeks. Additionally, one hundred forty-six (18.8%) and 31 (4%) pregnant women were emotionally and sexually/physically abused during their lifetime, respectively. A detail of the psychosocial risk of the study participants during pregnancy is available in Table 3.

### 3.4. Mental Health, Medical History, Substance Use, and Stressful Life Experience of the Study Participants

Twenty-eight (3.6%) pregnant women have confirmed mental illness, and 67 (8.6%) of the pregnant women had a family history of mental illness. Furthermore, 35.1% (273) and 41.7% of the pregnant women had financial stress and health risks, respectively. About two-thirds (61.4%) of the pregnant women have reported substance use, and the majority (288 (86.2%)) used alcohol. Mental health, medical history, substance use, and stressful life experience of the study participants are presented in Table 4.

### 3.5. Proportion and Determinants of Common Mental Disorders during Pregnancy

The current study showed that the prevalence of the common mental disorders among pregnant women was 18.1% (95% CI = 15.5, 21.0). The factors significantly associated with CMD include primary educational status of participants and their husbands, having a complication of current and previous pregnancies, undernutrition, high psychosocial risk during pregnancy, and having a relationship and legal problem (Table 5).

Pregnant women with primary education were 83% times less likely (AOR = 0.17, 95% CI = 0.06, 0.48) to have common mental disorders compared to those without formal education. Furthermore, women with unplanned pregnancies were about 2.5 times (AOR = 2.54, 95% CI = 1.26, 5.09) more likely to have maternal common mental disorders as compared with those women with a planned pregnancy. Pregnant women who reported not having complications during the current pregnancy were 89% (AOR = 0.11, 95% CI = 0.05, 0.21) less likely to have maternal common mental disorders, and pregnant women who reported experiencing complications during the previous delivery were three times (AOR = 3.38, 95% CI = 1.39, 8.18) more likely to have common mental disorders.

Moreover, the nutritional status of pregnant women was associated with common mental disorders. Undernourished pregnant women were twice (AOR = 2.14, 95% CI = 1.26, 3.81) more likely to have maternal common mental disorders than those who were not undernourished. Psychosocial risk of women showed a strong statistically significant association with maternal common mental disorders; pregnant women with high psychosocial risk were almost 21 times (AOR = 20.55, 95% CI = 9.69, 43.59) more likely to have maternal common mental disorders than those with low psychosocial risk.

Lastly, pregnant women who experienced relationship problems were two times (AOR = 2.06, 95% CI = 1.12, 3.79) more likely to have maternal common mental disorders compared with those who did not experience them; pregnant women who encountered legal problems were seven times (AOR = 7.22, 95% CI = 3.59, 14.53) more likely to have common mental disorders than their counterparts.

## 4. Discussion

In this study, nearly one in five pregnant women has common mental disorders. Primary education of pregnant women and their spouses, unplanned pregnancy, self-reported complication during current and previous pregnancy, nutritional status, psychosocial risk, and legal and relationship problems were significantly associated with common mental disorders.

The current finding revealed that the magnitude of CMD among pregnant women was 18.1% (95% CI = 15.5, 21.0). The finding of this study was consistent with a study conducted in Brazil (20.2%) [29], a systematic review in low- and low-middle-income countries (15.6%) [4], and a study in Uganda (16.7%) [30]. However, the current finding showed that the prevalence of common mental disorders during pregnancy was higher than that in studies conducted in Butajira (12%) [23] and Debre Tabor (11.8%) [31].

The possible reason for this difference is that the study in Butajira included only women in their third trimester. In contrast, all pregnant women, regardless of their trimester, were included in this study. Additionally, the variation may be due to the measurement tool used, study setting, and sample size, as it was a community-based study. The Edinburgh Postnatal Depression Scale was used to assess depression symptoms. The sample size is small in the study conducted in Debre Tabor, whereas this used an institutional-based study, large sample size, and SRQ-20 tool to assess CMD. Additionally, the finding was lower compared to studies conducted in Debre Birhan Town (45.2%) [32] and Southeast Ethiopia (35.8%) [21]. These variations may be due to differences in the study setting, as they were community-based cross-sectional studies and this is an institutional-based study.

In this study, pregnant women who had completed a primary level of education were 83% times less likely (AOR = 0.17) to have common mental disorders than those with no formal education. This finding was consistent with studies in Ethiopia and Southern Brazil, where being unable to read and write and lower educational levels were associated with CMD, respectively [21, 33]. This might be due to the low educational level often related to socioeconomic disadvantages such as low income, which is also a risk factor for mental health problems [33]. The other explanation might also be due to the reason that educated mothers have higher access to reproductive health information, and this, in turn, may reduce stress related to pregnancy [34]. In contrast, this study showed that women with a husband who completed a primary level of education were 11.13 times (AOR = 11.13) more likely to have common mental disorders than those with no formal education.

This study also found that pregnant women with complications during previous pregnancies were more than three (AOR = 3.38) times more likely to have common mental disorders than their counterparts. These findings were supported by studies conducted in Ethiopia [31, 32, 35]. The possible reason might be that pregnant women experience depressive symptoms, and having a complication during this period may make their condition worse [21]. This may be because of adverse life events affecting the person's mental health. Early detecting and appropriately managing pregnancy complications may reduce the risk of common mental disorders.

Additionally, women with high psychosocial risk were 20.55 (AOR = 20.55) times higher than those with low psychosocial risk. This was in line with studies conducted in lower-middle-income countries [4, 36–39]. This might be because pregnant women must share their pregnancy-related worries with their partners and social networks, which may help to reduce the risk of developing common mental disorder.

Moreover, pregnant women who had experienced relationship and legal problems were 2.06 (AOR = 2.06) and 7.22 (AOR = 7.22) times more likely to have common mental disorders than their counterparts. The finding was consistent with the studies conducted in South Africa, Tanzania, and Ethiopia [36, 40, 41]. This might be due to family-related factors; relationships with family members, especially with the husband, had valuable prognostic significance for antenatal mental health problems [42], implying that pregnant women with relationship problems and legal issues need support.

## 5. Conclusion and Recommendation

The finding of this study showed that nearly one in five pregnant women had common mental disorders. Participants and husbands' level of education, unplanned pregnancy, self-reported complication during the current and previous pregnancies, undernutrition, high psychosocial risk, and legal and relationship problems were the factors significantly associated with a common mental disorder. Antenatal care and mental health services must be integrated; screening for CMD should be part of the routine antenatal assessment and the need to refer the case to mental health services early. Professionals in antenatal care services must identify high-risk pregnant women and collaborate with the respective stakeholders to improve their mental health.

## Figures and Tables

**Table 1:** Sociodemographic characteristics of the study participants in North Wollo Zone, Amhara Region, Northeast Ethiopia (2022) (*n* = 777).

Variables	Frequency	Percentage (%)
Age		
<20	37	4.8
21–34	585	75.3
>34	155	19.9
Residence		
Rural	482	62.0
Urban	295	38.0
Religion		
Orthodox	507	65.3
Muslim	220	28.3
Protestant	50	6.4
Marital status		
Married	613	78.9
Single	94	21.1
Other^∗^	70	9
Educational status of women		
No formal education	326	42
Primary and secondary	129	16.6
College and above	322	41.4
Occupational status		
Housewife	271	34.9
Governmental employee	170	21.9
Merchant	154	19.8
Farmer	134	17.2
Unemployed	48	6.2
Husband's educational status		
No formal education	292	37.6
Primary and secondary	71	9.1
College and above	414	53.3
Husband's occupational status		
Farmer	195	25. 1
Merchant	254	32.7
Governmental employee	272	35.0
Unemployed	56	7.2

N.B.: ^∗^divorced, widowed, and separated.

**Table 2:** Obstetric characteristics of the study participants in North Wollo Zone, Amhara Region, Northeast Ethiopia (2022) (*n* = 777).

Variables	Frequency	Percentage (%)
Total number of pregnancies		
Primigravida	263	34
Multigravida	514	66
Gestational age at interview		
First trimester	44	5.7
Second trimester	316	40.7
Third trimester	417	53.7
MUAC measurement		
Less than 23 cm	251	32.3
23 cm and above	526	67.7
Current pregnancy complications		
Yes	123	15.8
No	654	84.2
History of past pregnancy complications		
Yes	128	16.5
No	386	49.7
History of abortion		
Yes	57	11.1
No	457	88.9
History of stillbirth		
Yes	18	3.5
No	496	96.5
Type of complications in the past pregnancy		
Spontaneous abortion	57	44.5
Gestational DM	20	15.6
Hypertension disorder	36	28.1
Vaginal bleeding	3	2.3
Edema of the hand and feet	6	4.6
Uterine rupture before birth	6	4.6
Is pregnancy planned		
Yes	683	87.9
No	94	12.1

**Table 3:** Psychosocial risk of the study participants in North Wollo Zone, Amhara Region, Northeast Ethiopia (2022) (*n* = 777).

Variables	Frequency	Percentage (%)
Psychosocial risk during pregnancy		
High	84	10.8
Low	693	89.2
History of being worried, miserable, or depressed for more than 2 weeks		
No	701	90.2
Yes	76	9.8
Seriously interfere with your work or your relationships with friends and family?		
Very much	0	0
Quite a lot	9	11.8
Somewhat	30	39.5
A little	21	27.6
Not at all	16	21.1
Lead you to seek professional help		
No	62	81.6
Yes	14	18.4
Is your relationship with your partner emotionally supportive?		
Very much	234	30.1
Quite a lot	159	20.5
Somewhat	143	18.4
A little	119	15.3
Not at all	88	11.3
Have you had any stresses, changes, or losses in the last 12 months?		
No	708	91.1
Yes	69	8.9
How distressed were you by these stresses, changes, or losses?		
Very much	19	27.5
Quite a lot	22	31.9
Somewhat	3	4.3
A little	22	31.9
Not at all	3	4.3
Would you generally consider yourself worried		
Very much	13	1.7
Quite a lot	13	1.7
Somewhat	74	9.5
A little	238	30.6
Not at all	439	56.5
Do you become upset if you do not have order in your life?		
Very much	7	0.9
Quite a lot	27	3.5
Somewhat	198	25.5
A little	259	33.3
Not at all	286	36.8
Do you feel you will have people you can depend on for support with your baby		
Very much	55	7.1
Quite a lot	99	12.7
Somewhat	256	32.9
A little	176	22.7
Not at all	191	24.6
When you were growing up, did you feel your mother was emotionally supportive of you?		
Very much	366	47.1
Quite a lot	190	24.5
Somewhat	22	2.8
A little	74	9.5
Not at all	121	15.6
Have you been emotionally abused when you were growing up		
No	631	81.2
Yes	146	18.8
Have you ever been sexually or physically abused		
No	746	96.0
Yes	31	4.0

**Table 4:** Mental health status, medical history, substance use, and stressful life experience of the study participants in North Wollo Zone, Amhara Regional State, Ethiopia (2022).

Variables	Frequency	Percentage (%)
Confirmed mental illness		
Yes	28	3.6
No	749	96.4
History of depression		
Yes	94	12.1
No	683	87.9
Family history of mental illness		
Yes	67	8.6
No	710	91.4
History of chronic illness		
Yes	81	10.4
No	696	89.6
Health risk		
Yes	324	41.7
No	453	58.3
Loss of loved ones		
Yes	427	55.0
No	350	45.0
Relationship problem		
Yes	156	20.1
No	621	79.9
Financial problems		
Yes	273	64.9
No	504	35.1
Legal issue		
Yes	101	13.0
No	676	87.0
Ever use of a substance		
Yes	300	38.6
No	477	61.4
Type of substances used		
Alcohol	288	86.2
Khat	28	8.4
Cigarette	14	4.2
Shisha	4	1.2
Use of a substance in the past three months		
Yes	220	73.3
No	80	26.7
Type of substance use in the past three months		
Alcohol	220	92.8
Khat	13	5.5
Cigarette	4	1.7

**Table 5:** Logistic regression analysis of maternal common mental disorders in North Wollo Zone, Amhara Regional State, Northeast Ethiopia (2022).

Variables	CMD		COR (95% CI)	AOR (95% CI)
	Yes	No		
Educational status of women				
No formal education	76	250	1	1
Primary and secondary	17	112	0.49 (0.28, 0.88)	0.17 (0.06, 0.48)^∗^
College and above	48	274	0.58 (0.39, 0.86)	0.54 (0.24, 1.22)
Educational status of husbands				
No formal education	59	233	1.47 (0.98, 2.17)	0.62 (0.27, 1.39)
Primary and secondary	21	50	2.43 (1.36, 4.33)	11.13 (4.18, 29.66)^∗∗^
College and above	61	353	1	1
Residence				
Urban	67	415	0.48 (0.33, 0.69)	0.73 (0.37, 1.44)
Rural	74	221	1	1
Gravidity				
Primigravida	10	253	0.12 (0.06, 0.22)	0.19 (0.02, 2.01)
Multigravida	131	382	1	1
Type of pregnancy				
Planned	103	580	1	1
Not planned	38	56	3.82 (2.41, 6.06)	3.82 (2.41, 6.06)^∗∗^
Current pregnancy complications				
No	77	577	0.12 (0.08, 0.19)	0.11 (0.05, 0.21)^∗∗^
Yes	64	59	1	1
History of past pregnancy complications				
No	85	301	1	1
Yes	46	82	7.15 (3.63, 14.05)	3.38 (1.39, 8.18)^∗∗^
Gestational age				
First trimester	2	42	0.24 (0.06, 1.02)	0.68 (0.12, 3.77)
Second trimester	70	246	1.44 (0.99, 2.08)	0.71 (0.37, 1.35)
Third trimester	69	348	1	1
MUAC				
Less than 23	62	189	1.86 (1.28, 2.69)	2.19 (1.26, 3.81)^∗^
23 and above	79	447	1	1
Psychosocial risk				
Low	82	611	1	1
High	59	25	17.59 (10.44, 29.62)	20.55 (9.69, 43.59)^∗∗^
Social support				
Poor	79	151	4.19 (2.61, 6.71)	1.45 (0.67, 3.10)
Moderate	33	253	1.04 (0.61, 1.77)	0.59 (0.29, 1.22)
Strong	29	232	1	1
Categories of stressors				
Health risk				
No	58	395	1	1
Yes	83	241	2.35 (1.62, 3.40)	1.68 (0.98, 2.86)
Relationship problem				
No	78	543	1	1
Yes	63	93	4.72 (3.17, 7.02)	2.06 (1.12, 3.79)^∗^
Loss of loved ones				
No	45	305	1	1
Yes	96	331	1.97 (1.34, 2.89)	1.17 (0.63, 2.18)
Financial stress				
No	73	431	1	1
Yes	68	205	1.96 (1.35, 2.84)	0.87 (0.47, 1.62)
Legal issue				
No	88	588	1	1
Yes	53	48	7.38 (4.70, 11.57)	7.22 (3.59, 14.53)^∗∗^

N.B.: ^∗^statistically significant at *p* < 0.005; ^∗∗^statistically significant at *p* < 0.0001; 1 = reference.

## Data Availability

The datasets used in this study are available from the corresponding author upon reasonable request.

## Abbreviations

ANC: Antenatal care
ANRQ: Antenatal Risk Questionnaire
AOR: Adjusted odds ratio
ASSIST: Alcohol, Smoking and Substance Involvement Screening Test
BMI: Body mass index
CMD: Common mental disorder
CI: Confidence interval
LMICs: Low- and middle-income countries
LTE-Q: Life-Threatening Events Questionnaire
MUAC: Mid-upper arm circumference
SRQ: Self-Reported Questionnaire
SPSS: Statistical Package for the Social Sciences
WHO: World Health Organization.
